# Anatomy of Subpancreatic Transverse Colon Vessel and Its Clinical Significance: An Observational Study

**DOI:** 10.3389/fsurg.2022.938223

**Published:** 2022-07-01

**Authors:** Jiankun Liao, Haiquan Qin, Li Wei, Zigao Huang, Linghou Meng, Wentao Wang, Xianwei Mo

**Affiliations:** ^1^ Division of Colorectal and Anal, Department of Gastrointestinal Surgery, Guangxi Medical University Cancer Hospital, Nanning, China; ^2^ Guangxi Clinical Research Center for Colorectal Cancer, Guangxi Medical University Cancer Hospital, Nanning, China; ^3^ Department of Human Anatomy, Guangxi Medical University, Nanning, China

**Keywords:** subpancreatic transverse colon vessel, transverse mesocolon, cadaver dissection, lymph node dissection, surgical anatomy

## Abstract

**Purpose:**

To observe and count the probability of presence and the anatomy of the vessel arising *via* the inferior margin of the pancreas and traveling within the transverse mesocolon, and analyze its clinical significance.

**Methods:**

Patients who underwent radical operation for transverse colon cancer or descending colon cancer from January 2020 to November 2021 and a nonspecific cadaver were included in this study. We observed and recorded intraoperatively for the probability of presence and the anatomy of the vessel arising *via* the inferior margin of the pancreas and traveling within the transverse mesocolon. And its property was determined by tissue slice.

**Results:**

A total of 84 patients were included, of which, the vessel was observed in 72 (85.7%) patients, and its property was confirmed by tissue slice of one patient after surgery. The vessel was also observed in a nonspecific cadaver. Originating from transverse pancreatic artery, often one, occasionally two, rarely three vessels arose *via* the inferior margin of pancreas and supplied the left transverse colon. Artery and vein parallel ran, and it was difficult to separate them due to their small diameter, but the vessels may thicken under certain conditions for increasing blood supply.

**Conclusion:**

The vessel, which is not yet reported and named in the literature, can be called the subpancreatic transverse colon vessel, which has a high probability of presence in humans and may be of great significance to human physiological anatomy, surgery, and oncology, and deserves recognition and attention from surgeons.

## Introduction

Lymphatic metastasis is a common way of malignant tumor metastases, and the degree of regional lymph node dissection (LND) is one of the independent factors affecting the survival and prognosis of patients (1–3).

It is well known that metastasis of tumor cells to lymph nodes (LNs) occurs through lymphatic drainage along tumor-feeding vessels. The main LN drainage in transverse colon cancer is along the middle colic artery (MCA). However, Aristotelis Perrakis et al. (4) have demonstrated the existence of infra-pancreatic lymph node region (ILR) and gastroepiploic arcade lymph node region (GLR) in patients with malignancy of transverse colon and of both flexures, which are not normal LN drainage regions. In the literatures, ILR and GLR were considered to be related to the existence of collateral vascular connections between the transverse colon and the gastric submental region and the subpancreatic region, but there were no studies on specific vessels (4–7).

With the assistance of 4K laparoscopy, which provides a higher-definition surgical view and enhances the surgeon’s grasp of anatomical levels and identification of subtle blood vessels, surgeons can clearly observe the vascular anatomy of the human body. Recently, a vessel was observed during operation in our clinical center, which arose *via* the inferior margin of the pancreas and traveled within the left transverse mesocolon, and jointly supplying blood to the transverse colon. The vessel observed in this study differed from the known vessels supplying transverse colon; by reviewing the relevant literatures, we found that it has not been previously reported or named in the literatures. In this study, we observed the characteristics of this vessel intraoperatively and analyzed its clinical significance in transverse colon blood supply, surgery, and oncology.

## Materials and Methods

The study was approved by the Ethics Committee of Guangxi Medical University Cancer Hospital. This was an observational study involving patients who underwent radical operation for transverse colon cancer or descending colon cancer in the Division of Colorectal and Anal of Guangxi Medical University Cancer Hospital from January 2020 to November 2021. Patients with clear intraoperative exposure of the inferior margin of pancreas that allowed observation of local anatomy were included, while patients with unclear or limited surgical field exposure that prevented observation of local anatomy were excluded.

### Clinical Assessment

Intraoperatively, when the gastrocolic ligament was incised, and the attachment of the transverse mesentery root to the pancreatic surface was separated so as to expose the inferior margin of pancreas, it was found that the vessel arose from the inferior margin of the pancreatic body and proceeded toward the transverse mesocolon. The presence of this vessel was observed and recorded intraoperatively. Moreover, the vessel of one patient was made into tissue slice and observed by a microscope to verify its property.

### Anatomical Assessment

In order to verify the actual existence of the vessel observed during operation, we selected a nonspecific cadaver specimen from the Department of Human Anatomy, Guangxi Medical University, to observe the anatomy of the vessel. The cadaver was formally registered as a specimen for teaching and research. The specimen was fixed with formaldehyde and its vessels were specially treated with a vascular contrast agent.

### Statistical Analysis

IBM SPSS 25.0 (IBM Corp, Armonk, NY, USA) software was used for statistical analysis. The chi-square test was used for comparison of enumeration data, and a *p *< 0.05 was defined as statistically significant.

## Results

A total of 84 patients, with a median age of 59 (29–82) years old, were included in the study. The characteristics and observations for the 84 patients are shown in Table 1. Of the 84 patients, 15 were diagnosed with transverse colon cancer and 69 were diagnosed with descending colon cancer and were performed radical transverse colectomy or left hemicolectomy, respectively.

**Table 1:** Characteristics and observations for the 84 patients.

	Patients (*n* = 84)
Gender	
Male	57 (67.9%)
Female	27 (32.1%)
Median age (range), (years)	59 (29–82)
Median BMI (range), (kg/m^2^)	22.54 (17.10–34.52)
Tumor location	
Transverse colon	15 (17.9%)
Descending colon	69 (82.1%)
Tumor size (cm)	
<5	12 (14.3%)
≥5	72 (85.7%)
Surgical mode	
Transverse colectomy	15 (17.9%)
Left hemicolectomy	69 (82.1%)
Subpancreatic transverse colon vessel	
Present	72 (85.7%)
Absent	12 (14.3%)
Number of vascular branches	
One	64 (76.2%)
Two	7 (8.3%)
Three	1 (1.2%)
Avascular	12 (14.3%)
Diameter of the vessel	
Thickening	14 (16.7%)
Normal	58 (69.0%)
Avascular	12 (14.3%)

*cm, centimeter, kg, kilogram, m, meter*.

### Clinical Outcome

**Figure 1:**
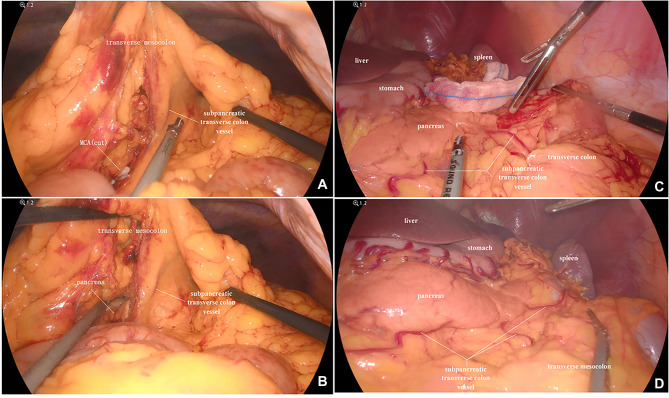
(**A**) A vessel was observed running through the transverse mesocolon, supplying blood to the transverse colon, except for the MCA. (**B**) The vessel which emanated from the inferior margin of the pancreas, supplied the transverse colon. (**C**) Two vessels were clearly observed. (**D**) Three vessels were clearly observed. MCA, middle colic artery.

**Figure 2:**
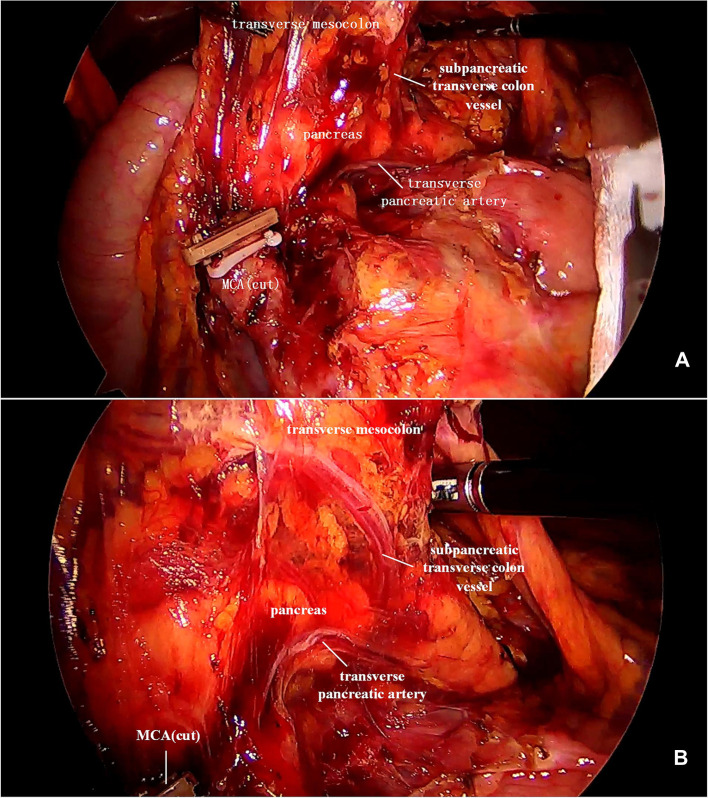
(**A**) This image showed a severed MCA, while the subpancreatic transverse colon vessel extending toward the transverse colon. (**B**) The subpancreatic transverse colon vessel originated from the TPA and supplied the left transverse colon. MCA, middle colic artery; TPA, transverse pancreatic artery.

**Figure 3:**
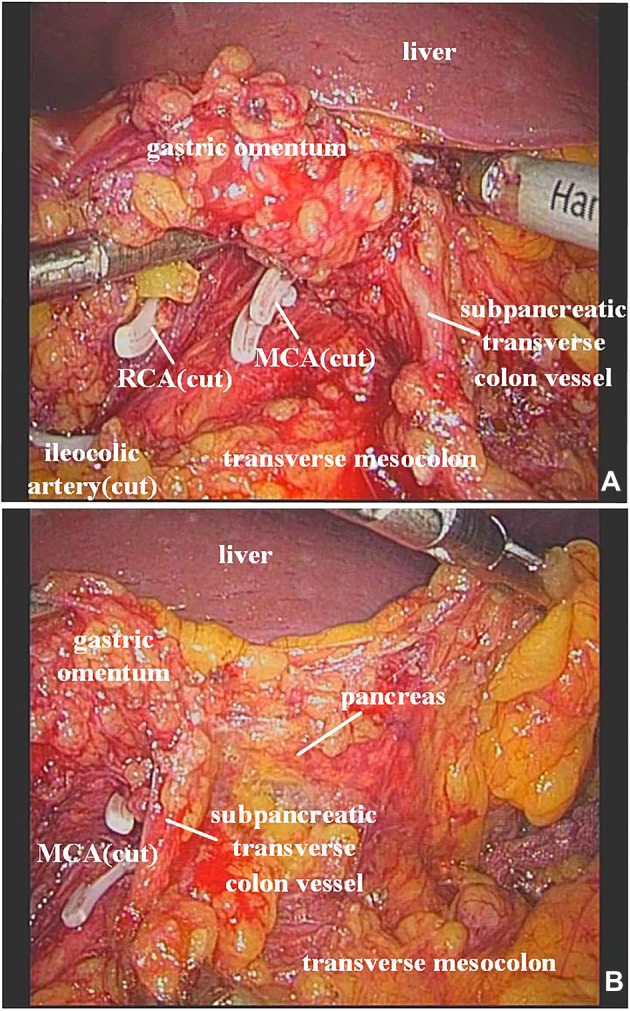
(**A**) It was observed that the ileocolic artery, RCA, and MCA were severed, and a thickened subpancreatic transverse colon vessel is naked. (**B**) The thickened subpancreatic transverse colon vessel emanated from the inferior margin of the pancreas, and supplied the transverse colon. RCA, right colic artery; MCA, middle colic artery.

In our study, the vessel which arose *via* the inferior margin of the pancreas and traveled within the left transverse mesocolon was observed in 72 (85.7%) patients. And a single vessel was observed in 64 (76.2%) patients, two vessels in 7 (8.3%) patients, and three vessels in 1 (1.2%) patient (Figure 1). Furthermore, sharp separation revealed that this vessel originated from transverse pancreatic artery (TPA), traveled within the left transverse mesocolon, converged into the vascular arch of the left transverse colon, and together supplied the left transverse colon (Figure 2). Furthermore, the diameter of this vessel was so small that the vessel could be cut off after coagulation with the ultrasonic knife; in the case of transverse colon tumors or with a large tumor volume, the vessel was involved in the tumor blood supply, and a thickening of the vessel diameter, approximately equal to the diameter of the MCA, could be observed (Figure 3).

### Analysis of Relation Between the Vessel and Its Diameter and Location of the Tumor

The presence of the vessel was independent of the location of the tumor (*p *> 0.05). However, in the presence of the vessel, the diameter of the vessel was correlated with the location of the tumor. The probability of vascular diameter thickening in the transverse colon tumor was greater than that in the left colon tumor (*p *< 0.05) (Table 2).

**Table 2:** Relation between the condition of subpancreatic transverse colon vessel and its diameter and location of the tumor.

		Location of the tumor	
		Transverse colon	Descending colon	*p*-value
Subpancreatic transverse colon vessel	Present	15	57	0.113
	Absent	0	12	
Diameter of the vessel	Thickening	8	6	0.001
	Normal	7	51	

### Histological Outcome

**Figure 4:**
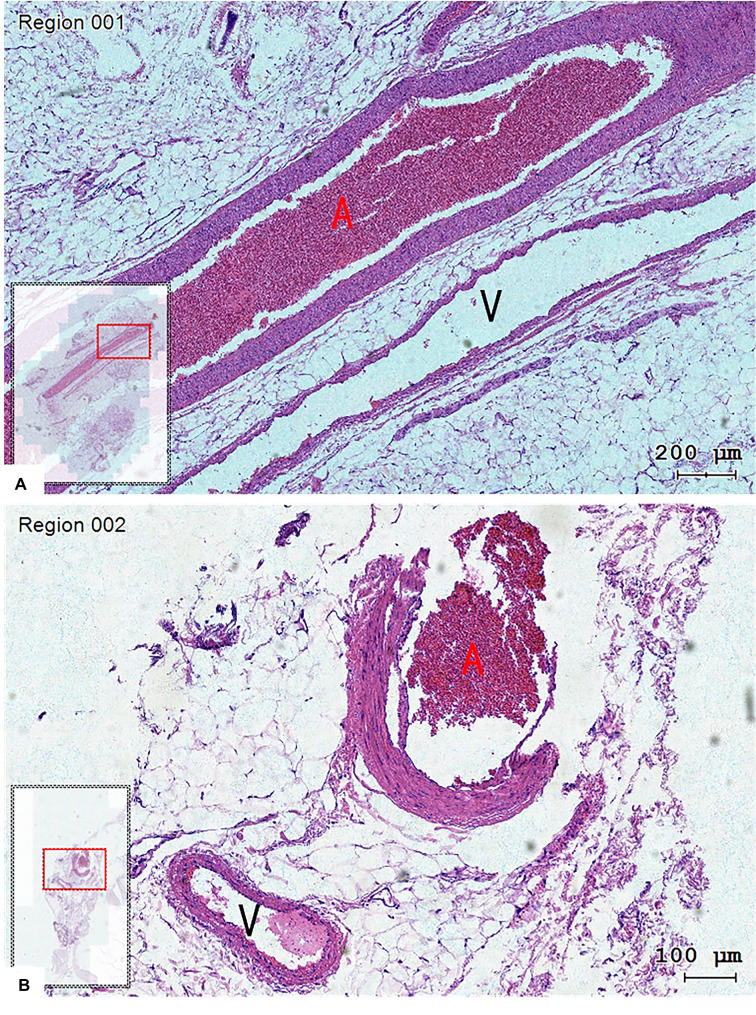
The structure of the arteriovenous wall could be observed in tissue slices, and the vein (V) run closely with the artery (A). (**A**) Coronal plane and (**B**) transverse plane. Hematoxylin-eosin staining, magnification 200× (**A**) and 100× (**B**).

The tissue slice made from the vessel of one patient was observed under a microscope, and the complete arteriovenous wall structure could be observed (Figure 4). The arteriovenous parallel running was observed on the coronal section.

### Anatomical Outcome

**Figure 5:**
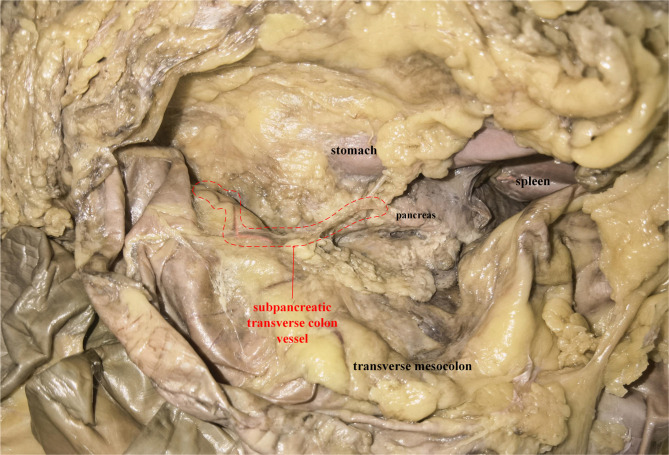
A plane view of pancreas-transverse mesocolon in a cadaver: the subpancreatic transverse colon vessel was circled (red dotted line). And the vessel was conducted with angiography prior to dissection. The artery was pink, while the vein was brown.

An adult male cadaver was included in this study, and the cause of death was not related to this study. After entering the transverse colon and pancreas plane according to the above steps, we observed a vessel originating from the TPA, arising *via* the inferior margin of the pancreas, and traveling within the transverse mesocolon (Figure 5). Arteriovenous parallel can be seen with the help of a vascular contrast agent, but due to the small diameter of the vessel, arteriovenous separation is not possible, and the perivascular fat of the vessel cannot be completely removed to fully reveal the vascular flow.

## Discussion

Central vascular ligation and LND have been generally accepted (8), but some studies (7, 9) had found that extra-regional LN metastases could exist in malignant tumors of the transverse colon, especially in patients with advanced cancers. Although the route of LN drainage is unclear, the presence of vascular connections between the transverse colon and pancreas is not surprising given the embryological background and the anatomic differentiation of the membrane between them (10). And lymphatic drainage along the vessels to infra-pancreatic LNs may explain the occurrence of these unconventional regional LN metastases.

In this study, the vessel could be clearly observed in 72 (85.7%) patients during operation, and the existence of the vessel was confirmed by autopsy in one cadaver, with arteriovenous parallel running and a small diameter. The autopsy revealed that the vessel originated from the TPA, and converged into the vascular arch of the left transverse colon, which together supply the left transverse colon. Consensus aside from the MCA, it has been reported (11) that accessory middle colic artery (AMCA) usually originates from the superior mesenteric artery (SMA) and a few from the inferior mesenteric artery (IMA), the abdominal aorta, or the intrinsic hepatic artery (IHA). It penetrates the dorsal aspect of the pancreas to supply the splenic flexure, and a small number of the AMCA may branch into the dorsal pancreatic artery (DPA) and the inferior pancreaticoduodenal artery (IPDA) to supply the pancreas (12). Also, Dionysios Venieratos (13) observed rare middle mesenteric artery (MMA) in a cadaveric specimen, and it originated from the abdominal aorta, in which two branches replace the posterior and anterior branches of the IPDA, supplying the head of the pancreas and the transverse colon. The vessel in our study is similar in location to the AMCA and MMA reported in the past. Although the origin and vascular pattern were not consistent, strictly speaking, the AMCA or MMA with a special pattern could not be ruled out. At present, this vessel, which is present in most individuals and has important implications, has not been reported and named, and it should deserve recognition and attention by clinicians. Based on the anatomic location and role of this vessel, we just call it the subpancreatic transverse colon vessel.

When the tumor is located in the transverse colon or descending colon, the presence of subpancreatic transverse colon vessel was 100% and 82.6%, respectively, which is no statistical difference. It shows that the presence of the vessel has nothing to do with the location of the tumor, the emergence of the vessel is normal, rather than pathological, but the study did not join the normal data by contrast, the credibility of the conclusion remains to be verified. Moreover, in the presence of subpancreatic transverse colon vessel, vascular diameter is related to the location of the tumor, and when the tumor is located in the transverse colon, the probability of vascular diameter thickening is greater. In our opinion, it should be related to the pathologic thickening and become a nutrient vessel of the tumor caused by the blood supply needs of the tumor, but this is only the result of intraoperative visual measurement, and the specific vascular diameter needs to be accurately measured in subsequent studies. It is safe to cut off the subpancreatic transverse colon vessel after coagulation with ultrasonic knife when the diameter of the vessel is not large. However, when the diameter of the vessel is thickened, it may be at risk of rebleeding if the vessel is cut off only after coagulation with an ultrasonic knife. In this case, the vessel should be ligated and then cut off to prevent rebleeding.

The vascular arches of the colon margin are prone to inadequate anastomosis at the ileocecal, splenic flexure, and sigmoid-rectal junction, creating a weak point of blood supply (14). In the absence of the left branch of the MCA, the ascending branch of the left colic artery (LCA) (15, 16), or the absence of the Riolan arterial arch (17, 18), or in the case of a verbose transverse colon with inadequate anastomosis of the vascular arch, it may lead to a weak blood supply to the splenic flexure, which is called Griffiths critical point (19). Clinical reports of anastomotic leakage caused by insufficient blood supply of splenic flexure are rare. In this study, there was no anastomotic leakage occurring in 84 patients. Even in the previous radical surgery for bi-primary cancer of ascending colon and sigmoid colon, after the removal of MCA and IMA, the anastomotic stoma was established in the splenic flexure, and anastomotic leakage did not occur due to anastomotic ischemia after surgery. We consider that the subpancreatic transverse colon vessel may play an important role in ensuring blood supply. If the presence of the subpancreatic transverse colon vessel and its importance are not realized during intraoperative separation of the pancreas from the transverse mesocolon and this vessel is damaged, it may affect the anastomotic blood supply and lead to unnecessary resection of more intestinal segments. Therefore, we should identify and protect this vessel during surgery to ensure adequate blood supply to the proximal end of the anastomosis.

Tumor angiogenesis is not only a prerequisite for tumor growth, but also an important factor in promoting tumor metastasis. As the concept of “tumor angiogenesis” was first introduced by Algire (20) in 1945, there is now evidence (21) that tumor neovascularization is essential for the proliferation and growth of the primary tumor itself, and is also necessary for tumor invasion and metastasis.

Lymphatic metastasis is a common mode of metastasis in malignant tumors, and lymphatic drainage mostly follows nutrient vessels of tumors (22). The degree of LN metastases and surgical dissection are closely related to the prognosis of patients (23, 24). When there is a tumor occurs, the transverse colon has a complex anatomy compared with other parts of colon. In addition to lymphatic drainage along the tumor-feeding vessels to the mesangial LNs, abnormal lymphatic drainage—extracolonic LNs have already been found (4, 7). In addition, previous studies have also found that transverse colon cancer tends to have a worse prognosis than tumors elsewhere in the colon (25). The inferior pancreatic margin LNs belong to the 18th group, which is not part of the routine LND for colon cancer. In contrast, the subpancreatic transverse colon vessel provided a pathway for tumor invasion and metastasis, and especially in large transverse colon tumors, vascular diameter is enlarged for increasing blood flow, while also increasing the chance for tumor invasion and metastasis. Nowadays, the resection of the transverse mesocolon to its root is already recommended in routine oncologic practice, but the standard of dissection along the inferior margin of the pancreas, and even the posterior part of the pancreas, remains to be determined. Thereout, the presence of the subpancreatic transverse colon vessel provides the basis for future research.

In addition, the identification of the subpancreatic transverse colon vessel is important not only to surgical procedures but also to vascular interventions for tumors, such as transcatheter arterial chemoembolization (TACE) (26).

To be honest, there are significant limitations to our study. Firstly, this study was only a clinical observational study adhering to medical ethics which presented preliminary vascular observational results *via* radical operation for colon cancers according to the standard operating procedure and excision range, so it was unable to show the anatomy of subpancreatic transverse colon vessel in detail, especially its origin during operation. However, in combination with tissue section and autopsy, we objectively confirmed the existence and anatomy of the vessel in this study. Secondly, we only observed this vessel in one cadaver specimen, which lacks comprehensive vascular data. Furthermore, the study should be supplemented with results of lymph node metastases to support the effect of vascular presence on lymphatic diffusion. In order to show sufficient vascular evidence to clinicians, future research will continue from the above limitations.

## Conclusion

According to our study, 72 (85.7%) patients had the subpancreatic transverse colon vessel that appeared to be of great significance in anatomy, physiology, surgery, and oncology, and this requires surgeons to be aware of its importance and know how to identify and correctly manage the subpancreatic transverse colon vessel during operation. Meanwhile, we also encourage more surgeons to explore the anatomy and clinical significance of subpancreatic transverse colon vessel.

## Data Availability

The original contributions presented in the study are included in the article/Supplementary Material, further inquiries can be directed to the corresponding author/s.

## References

[1] Hong K Lee S, and Moon H. (2011). Lymph node ratio as determined by the 7th edition of the American Joint Committee on Cancer staging system predicts survival in stage III colon cancer. J Surg Oncol. 103 (5), 406–10. 10.1002/jso.21830 21400524

[2] Ramos-Esquivel A Juárez M González I Porras J, and Rodriguez L. (2014). Prognosis impact of the lymph node ratio in patients with colon adenocarcinoma: a single-centre experience. J Gastrointest Cancer. 45 (2), 133–6. 10.1007/s12029-013-9576-5 24382601

[3] Schumacher P Dineen S Barnett C Fleming J, and Anthony T. (2007). The metastatic lymph node ratio predicts survival in colon cancer. Am J Surg. 194 (6), 827–31; discussion 831–2. 10.1016/j.amjsurg.2007.08.030 18005779

[4] Perrakis A Weber K Merkel S Matzel K Agaimy A Gebbert C (2014). Lymph node metastasis of carcinomas of transverse colon including flexures. Consideration of the extramesocolic lymph node stations. Int J Colorectal Dis. 29 (10), 1223–9. 10.1007/s00384-014-1971-2 25060216

[5] Bertelsen C Bols B Ingeholm P Jansen J Jepsen L Kristensen B (2014). Lymph node metastases in the gastrocolic ligament in patients with colon cancer. Dis Colon Rectum. 57 (7), 839–45. 10.1097/DCR.0000000000000144 24901684

[6] Hohenberger W Weber K Matzel K Papadopoulos T, and Merkel S. (2009). Standardized surgery for colonic cancer: complete mesocolic excision and central ligation – technical notes and outcome. Colorectal Dis. 11 (4), 354–64; discussion 364–5. 10.1111/j.1463-1318.2008.01735.x 19016817

[7] Yuksel B Er S Çetinkaya E, and Aşlar A. (2021). Does transverse colon cancer spread to the extramesocolic lymph node stations? Acta Chir Belg. 121 (2), 102–8. 10.1080/00015458.2019.1689642 31701816

[8] West N Kobayashi H Takahashi K Perrakis A Weber K Hohenberger W (2012). Understanding optimal colonic cancer surgery: comparison of Japanese D3 resection and European complete mesocolic excision with central vascular ligation. J Clin Oncol. 30 (15), 1763–9. 10.1200/JCO.2011.38.3992 22473170

[9] Stelzner S Hohenberger W Weber K West N Witzigmann H, and Wedel T. (2016). Anatomy of the transverse colon revisited with respect to complete mesocolic excision and possible pathways of aberrant lymphatic tumor spread. Int J Colorectal Dis. 31 (2), 377–84. 10.1007/s00384-015-2434-0 26546443

[10] Matsuda T Iwasaki T Sumi Y Yamashita K Hasegawa H Yamamoto M (2017). Laparoscopic complete mesocolic excision for right-sided colon cancer using a cranial approach: anatomical and embryological consideration. Int J Colorectal Dis. 32 (1), 139–41. 10.1007/s00384-016-2673-8 27714518

[11] Murono K Miyake H Hojo D Nozawa H Kawai K Hata K (2020). Vascular anatomy of the splenic flexure, focusing on the accessory middle colic artery and vein. Colorectal Dis. 22 (4), 392–8. 10.1111/codi.14886 31650684

[12] Ito K Takemura N Inagaki F Mihara F Kurokawa T, and Kokudo N. (2019). Arterial blood supply to the pancreas from accessary middle colic artery. Pancreatology. 19 (5), 781–5. 10.1016/j.pan.2019.05.458 31164320

[13] Venieratos D Tsoucalas G, and Panagouli E. (2018). A rare branching pattern of a middle mesenteric artery supplying the head of the pancreas and the transverse colon. Acta Med Acad. 47 (2), 199–203. 10.5644/ama2006-124.232 30585072

[14] Coffey J. (2013). Surgical anatomy and anatomic surgery – clinical and scientific mutualism. Surgeon. 11 (4), 177–82. 10.1016/j.surge.2013.03.002 23597667

[15] Cirocchi R Randolph J Cheruiyot I Davies J Wheeler J Lancia M (2020). Systematic review and meta-analysis of the anatomical variants of the left colic artery. Colorectal Dis. 22 (7), 768–78. 10.1111/codi.14891 31655010

[16] Fukuoka A Sasaki T Tsukikawa S Miyajima N, and Ostubo T. (2017). Evaluating distribution of the left branch of the middle colic artery and the left colic artery by CT angiography and colonography to classify blood supply to the splenic flexure. Asian J Endosc Surg. 10 (2), 148–53. 10.1111/ases.12349 28008722

[17] Huang J Zhou J Wan Y Lin Y Deng Y Zhou Z (2016). Influences of inferior mesenteric artery types and Riolan artery arcade absence on the incidence of anastomotic leakage after laparoscopic resection of rectal cancer. Zhonghua Wei Chang Wai Ke Za Zhi. 19 (10), 1113–18. 27781246

[18] Karatay E Ekci B, and Javadov M. (2020). Should surgeons evaluate the anatomy of Drummond marginal artery and Riolan’s arch preoperatively? Surg Technol Int. (2020) 37:102–6. 32819026

[19] Griffiths J. (1956). Surgical anatomy of the blood supply of the distal colon. Ann R Coll Surg Engl. 19 (4), 241–56. 13363265 PMC2378072

[20] Algire G, and Legallais F. (1948). Growth and vascularization of transplanted mouse melanomas. Ann N Y Acad Sci. 4, 159–70. 18862169

[21] Jiang X Wang J Deng X Xiong F Zhang S Gong Z (2020). The role of microenvironment in tumor angiogenesis. J Exp Clin Cancer Res. 39 (1), 204. 10.1186/s13046-020-01709-5 32993787 PMC7526376

[22] Spasojevic M Stimec B Dyrbekk A Tepavcevic Z Edwin B Bakka A (2013). Lymph node distribution in the d3 area of the right mesocolon: implications for an anatomically correct cancer resection. A postmortem study. Dis Colon Rectum. 56 (12), 1381–7. 10.1097/01.dcr.0000436279.18577.d3 24201392

[23] Berger A Sigurdson E LeVoyer T Hanlon A Mayer R Macdonald J (2005). Colon cancer survival is associated with decreasing ratio of metastatic to examined lymph nodes. J Clin Oncol. 23 (34), 8706–12. 10.1200/JCO.2005.02.8852 16314630

[24] Chang G Rodriguez-Bigas M Skibber J, and Moyer V. (2007). Lymph node evaluation and survival after curative resection of colon cancer: systematic review. J Natl Cancer Inst. 99 (6), 433–41. 10.1093/jnci/djk092 17374833

[25] Sjo O Lunde O Nygaard K Sandvik L, and Nesbakken A. (2008). Tumour location is a prognostic factor for survival in colonic cancer patients. Colorectal Dis. 10 (1), 33–40. 10.1111/j.1463-1318.2007.01302.x 17672872

[26] Yun X Meng H Zhou A Jia J, and Qian W. (2021). Efficacy of transcatheter arterial chemoembolization combined with capecitabine and cetuximab in the treatment of colorectal cancer with liver metastasis. J BUON. 26 (3), 1002–8. 34268965

